# Faecal Steroid Metabolite Profiles and Reproductive Outcomes in Domestic Cat (*Felis catus*)

**DOI:** 10.3390/ani16162523

**Published:** 2026-08-13

**Authors:** Ksenia A. Volobueva, Mariya N. Erofeeva, Galina S. Alekseeva, Mariya D. Kim, Aleksandra S. Fetisova, Polina S. Zhuravleva, Sergey V. Naidenko

**Affiliations:** Severtsov Institute of Ecology and Evolution, Russian Academy of Sciences, 119071 Moscow, Russia; erofeevamariya@yandex.ru (M.N.E.); gal.ser.alekseeva@gmail.com (G.S.A.); mariya-d-kim@yandex.ru (M.D.K.); alexfetisova2017@yandex.ru (A.S.F.); klyuchnikova.polia@yandex.ru (P.S.Z.); snaidenko@mail.ru (S.V.N.)

**Keywords:** glucocorticoids, sex steroids, endocrine regulation, maternal physiology, reproductive success, kitten viability, non-invasive monitoring, Felinae

## Abstract

Research on endocrine patterns associated with reproduction and lactation in felids has focused mainly on rare or endangered species and remains limited in the domestic cat (*Felis catus*). Using a non-invasive longitudinal approach, we measured faecal glucocorticoid, progesterone, and testosterone metabolites (fGCm, fPm, and fTm) in nine females before mating and during three months of lactation, examining associations with litter characteristics and kitten survival. Lactational concentrations were expressed relative to each female’s median baseline, while baseline concentrations were analysed separately. Relative fGCm concentrations were negatively associated with the number of surviving kittens but not with litter losses. Relative fPm concentrations were positively associated with both fGCm and fTm, whereas the fGCm–fTm relationship did not reach statistical significance. Relative fPm concentrations were lower in the third than in the first month of lactation, while fTm showed a similar decreasing tendency. Baseline fGCm and fPm were not associated with reproductive parameters. Median baseline fTm showed a negative, non-significant relationship with first-month kitten survival. These findings indicate distinct temporal patterns and associations among steroid metabolites, while the relationship between baseline fTm and kitten survival requires validation in larger datasets.

## Simple Summary

Hormones regulate many physiological processes, including reproduction and lactation. However, these endocrine changes remain insufficiently understood in domestic cats. In this study, we measured faecal metabolites of glucocorticoids, progesterone, and testosterone in nine female domestic cats before mating and during three months of lactation. Lactational concentrations were expressed relative to each female’s median baseline concentration, and we examined their relationships with litter characteristics and kitten survival. Higher relative glucocorticoid metabolite concentrations during lactation were associated with fewer surviving kittens, although they were not associated with litter losses. Relative progesterone metabolite concentrations were lower in the third than in the first month of lactation. Testosterone metabolites were also lower in the third than in the first month, although the overall difference among lactation periods was not statistically significant. Baseline glucocorticoid and progesterone metabolite concentrations were not related to reproductive outcomes. Median baseline testosterone metabolite concentrations showed a negative relationship with first-month kitten survival, but this did not reach statistical significance. These findings characterise maternal endocrine changes in domestic cats and highlight hormone patterns that may be relevant to early kitten survival.

## 1. Introduction

Hormonal regulation plays a key role in coordinating physiological and behavioural processes associated with reproduction, maternal investment and offspring survival. Lactation is one of the most energetically demanding stages [1] and requires substantial physiological adjustments mediated by the endocrine system. During pregnancy, the corpora lutea contribute primarily to progesterone production during the first half of gestation, whereas the placenta becomes an important steroidogenic source during the second half [2]. Following parturition, the maternal endocrine system undergoes substantial reorganisation. Steroid and peptide hormones, including reproductive steroid hormones (progesterone, oestradiol), pituitary hormones (oxytocin, prolactin), and glucocorticoids (cortisol, corticosterone), are involved in the regulation of lactation and maintenance of maternal physiological balance. Additionally, testosterone may also influence lactation and maternal physiology [3]. In female mammals, it is secreted in small amounts by the ovaries and adrenal glands, while the adrenal contribution occurs mainly through dehydroepiandrosterone (DHEA) and androstenedione, which are converted to testosterone in peripheral tissues [4]. Along with short-term hormonal changes accompanying reproduction, baseline endocrine levels may also affect maternal physiology, energy balance and offspring survival [5,6]. Through their effects on energy allocation, parental behaviour and lactation, hormones mediate the balance between maternal self-maintenance and investment in offspring [7].

Despite extensive research on the hormonal profiles of various felid species, much of the focus has been directed towards rare and endangered species [8]. The question of the endocrine mechanisms regulating lactation in more common species of this family still remains open, as previous studies have primarily considered seasonal dynamics of sex hormones [9,10] and changes in cortisol levels due to external influences [11,12,13]. Some reproductive and lactation features have been described in certain species, for instance, in the Eurasian lynx (*Lynx lynx*), including strictly seasonal breeding [14] and persistently high progesterone concentrations not only during pregnancy but also throughout lactation, a phenomenon considered atypical for felids [15]. The domestic cat (*Felis catus* Linnaeus, 1758), a member of the subfamily Felinae, has received little attention in this context. This species is widely distributed across the globe and exhibits a remarkable reproductive capacity, with females capable of producing up to three litters per year, and litter size may reach up to eight kittens [16]. This feature of the reproductive physiology of domestic cats makes them a suitable model for investigating the factors affecting both prenatal and postnatal offspring development [1,17]. Understanding how hormonal fluctuations during lactation may be related to maternal physiological condition and offspring survival is vital for enhancing our comprehension of lactation and also holds significant implications for conservation efforts targeting wild felid populations. By elucidating the endocrine mechanisms underlying lactation in a widely available and well-studied species, strategies can be developed to improve reproductive success and offspring survival in wild felids.

Over the last few decades, non-invasive research methods have been frequently used to estimate changes in the physiological state of the organism [12,18,19]. These methods have the advantage of providing reliable information about the animal’s condition without direct interaction with the individual and without affecting its behaviour and physiology [20]. One of the popular directions among non-invasive methods for investigating the hormonal status of animals is the use of faeces to analyse hormone levels, as this method provides “pooled” information over 12–24 h (specifically in felids), unlike, for example, blood hormone analysis, where data correspond to a shorter time interval [21,22]. Considering that in felids, most (about 90%) of steroid hormone metabolites are excreted in faeces [23], this approach is the most convenient for studying their dynamics in this group of animals.

The aim of this study was to evaluate changes in the concentrations of the main steroid hormones (cortisol, progesterone, and testosterone) throughout lactation in female domestic cats and to examine their associations with reproductive parameters, including offspring survival and litter losses, as well as the relationships among the hormones themselves. In addition, we assessed whether baseline hormone concentrations measured before mating were associated with subsequent reproductive outcomes. We expected that glucocorticoid concentrations would be related to litter size due to the high energetic demands associated with rearing offspring [1]. Since progesterone may contribute to the regulation of lactation and maternal physiology in mammals [24,25], we also hypothesised that progesterone concentrations would vary throughout lactation and would be associated with the number of surviving kittens. Given the potential role of testosterone in modulating maternal physiology and reproductive outcomes [26,27], we hypothesised that elevated testosterone concentrations would be associated with lower offspring survival and increased litter losses.

## 2. Materials and Methods

### 2.1. Study Site

The study was carried out at the Joint Usage Centre “Live Collection of Wild Mammals” of the Severtsov Institute of Ecology and Evolution, Russian Academy of Sciences (biological station “Tchernogolovka”), located 50 km northeast of Moscow (56°00′ N, 38°22′ E). The study was conducted from 20 February 2022 to 17 September 2022. The temperature range during the experiment varied from −16 °C (11 March 2022) to +33 °C (26 August 2022); the average temperature was +11 °C.

### 2.2. Study Animals

A total of nine sexually mature domestic cat individuals were used in the experiment. The mean body mass of females before mating was 3.34 ± 0.1 (hereafter M ± SE) kg. Litter size ranged from 4 to 8 kittens after parturition (mean number of kittens per litter: 5.22 ± 0.47). The number of surviving kittens ranged from 2 to 6 by the end of the first month of lactation (4.33 ± 0.44), and from 2 to 5 by the end of the second and third months of lactation (3.89 ± 0.35). Kitten mortality occurred primarily during the first days of lactation, reaching 17% by the end of the first month, with an additional 10% observed by the end of the second month, and 0% by the end of the third month (Table 1).

### 2.3. Husbandry Conditions

During the experiment, the animals were housed outdoor individually, except for the period when mating took place and when females raised the kittens, under natural light and temperature conditions in an open-air enclosure complex. The cats were kept in enclosures of 6 m^2^ (2 × 3 m). All enclosures had a height of 2 m and were constructed with metal mesh walls and a metal mesh roof. Inside each enclosure, there was an artificial shelter: a wooden box measuring 0.9 × 0.6 × 0.5 m covered with sheet iron on top. The daily diet during the study consisted of approximately 300 g of chicken meat with bones or minced chicken meat with additional vitamins. The diet of lactating females was approximately 1.5 times larger. Animals were provided with water ad libitum.

### 2.4. Sample Collection

Baseline hormone levels were measured in all nine domestic cats based on 1–8 faecal samples per individual collected since 20 February 2022. The variation in the number of samples reflected individual differences in the onset of oestrus and the date at which each female was mated. During lactation, faecal samples were collected from each individual once per week in the morning. The first sample represented the earliest available postpartum sample (point 0), followed by weekly collections up to the 12th week postpartum. Each sample aliquot was packed in a separate plastic bag, labelled with the animal’s name, date and time of collection. The samples were frozen and stored at −18 °C until extraction.

### 2.5. Steroid Extraction and Analysis

The steroid extraction was conducted using a previously described method [28]. Briefly, 0.1 g of faeces was weighed in an Eppendorf tube on an Ohaus Pioneer scale (Ohaus Europe GmbH, Nänikon, Switzerland), and 0.9 mL of 90% methanol was added. The tubes were shaken for 30 min using an “Ecros” shaker (ECROSKHIM, Saint-Petersburg, Russia). The tubes were then centrifuged for 10 min at 4000 rpm using the Eppendorf MiniSpin centrifuge (Eppendorf, Hamburg, Germany). Using a reusable pipette, 400 µL of supernatant was withdrawn into a separate tube, and 400 µL of distilled water was added. These extracts were labelled and stored at −18 °C until measurements were taken. Hormone concentrations were measured by ELISA (see below) and recalculated on a dry faeces mass basis. We dried an aliquot of the sample in a desiccator cabinet at 80 °C until a constant weight was achieved and then calculated the moisture percentage.

Cortisol levels in domestic cats were determined using the “ImmunoFA_Cortisol” kits (“Immunotech,” Moscow, Russia). The test sensitivity was 32.6 ng/g. The cross-reactivity of the antibodies used for cortisol was 6.0% for prednisolone, 0.9% for 11-deoxycortisol, 0.6% for corticosterone, and less than 0.08% for other steroids. Preliminary validation of the antibodies used for measuring glucocorticoid levels in domestic cats using non-invasive methods was conducted previously [29].

Progesterone levels were determined using the “ImmunoFA_PG” kits (“Immunotech,” Moscow, Russia). The test sensitivity was 45.3 ng/g. The cross-reactivity of the antibodies used for progesterone was 0.07% for deoxycorticosterone, 0.03% for corticosterone, 0.01% for cortisol, and less than 0.01% for other steroids. Validation of the antibodies used for measuring progesterone levels in domestic cats using non-invasive methods was performed previously [29].

Testosterone levels were determined using the “ImmunoFA_TS” kits (“Immunotech,” Moscow, Russia). The test sensitivity was 20.8 ng/g. The cross-reactivity of the antibodies used for testosterone with other steroids was 9% for 5a-dihydrotestosterone, 1% for 11-hydroxytestosterone and 5-androstane-3,17-diol, 0.66% for progesterone, and less than 0.08% for other steroids. Preliminary validation of the antibodies used for measuring testosterone levels in domestic cats using non-invasive methods was performed previously [29].

Measurements were taken in duplicate, determining the coefficient of variation (CV). If the CV was greater than 10%, the measurements were repeated; if the CV was less than 10%, the mean value was accepted for further analysis. The intra-assay CV for cortisol was 2.67 ± 0.35% (*n* = 441), and the inter-assay CV for a control sample with a concentration of 12.5 ng/mL, measured on different plates, was 7.7% (*n* = 17). The intra-assay CV for progesterone was 3.65 ± 0.60% (*n* = 441), and the inter-assay CV for a control sample with a concentration of 0.625 ng/mL, measured on different plates, was 15.42% (*n* = 16). The intra-assay CV for testosterone was 2.74 ± 0.3% (*n* = 441), and the inter-assay CV for a control sample with a concentration of 0.544 ng/mL, measured on different plates, was 9.21% (*n* = 21).

The use of antibodies to steroids such as cortisol, progesterone, and testosterone in the analysis allows measuring not only the concentration of the hormone itself, but also the concentration of hormone metabolites, their conjugates and a number of other compounds due to the cross-reactivity of the antibodies used [30]. For the convenience in presenting the materials, we used the terms “fGCm” (faecal glucocorticoid metabolite), “fPm” (faecal progesterone metabolite) and “fTm” (faecal testosterone metabolite) to refer to the entire group of compounds that bind to the antibodies against these steroid hormones.

To analyse steroid hormone concentrations, we used both the absolute concentration values and the relative deviations from individual median baseline levels in percent (relative concentration). Individual median baseline hormone levels were measured before mating, and percentage changes were calculated for each stage of lactation (first, second, and third month). This dual approach was adopted to account for the substantial inter-individual variability in hormone concentrations, which can occur even among individuals of the same sex [31,32].

### 2.6. Statistical Analysis

Statistical analyses were performed using R version 4.3. using R version 4.3.2 (R Core Team, Vienna, Austria). Model assumptions were assessed according to model type. For linear models (LMs) and linear mixed-effects models (LMMs), the normality and homoscedasticity of residuals were evaluated using the Shapiro–Wilk test and visual inspection of Q–Q plots and residual-versus-fitted plots. Variables were natural-log-transformed when necessary to improve model assumptions. For binomial generalised linear models (GLMs), overdispersion was assessed.

Associations between baseline steroid hormone concentrations and reproductive outcomes were analysed separately for fGCm, fPm, and fTm. Litter size at birth was analysed using linear models (LMs) implemented in the stats package. Kitten survival to the end of the first month was analysed using binomial generalised linear models (GLMs), with the numbers of surviving and lost kittens included jointly as the response variable. Pearson’s correlation analysis was additionally used to examine the relationship between baseline fTm concentrations and the number of kittens surviving to the end of the first month.

To analyse hormonal variation during lactation, linear mixed-effects models (LMMs) were fitted using the lme4 package. Individual identity of female cats (ID) was included as a random effect to account for repeated measurements. For each hormone, a global model was constructed including the following fixed effects: concentrations of the other steroid hormones, lactation period (month), number of surviving kittens and litter losses. Model selection was performed using an information-theoretic approach based on Akaike’s Information Criterion corrected for small sample size (AICc), implemented in the MuMIn package. Candidate models were generated using the dredge function [33] with REML set to FALSE. Models with ΔAICc < 2 were retained. When multiple models met this criterion, model averaging was applied. Parameter estimates, standard errors, and significance levels were obtained using conditional model averaging. The relative importance of predictors was assessed using the sum of Akaike’s weights. For each model, 95% confidence intervals were calculated.

Residuals of the best-supported models, as well as all models within ΔAICc < 2, were visually inspected using Q–Q plots to assess normality. Minor deviations from normality were considered acceptable.

To specifically assess temporal changes in hormone concentrations, additional linear mixed-effects models including only lactation period (time) as a fixed effect and individual identity as a random effect were constructed.

Post hoc pairwise comparisons between lactation periods were performed using Tukey-adjusted estimated marginal means implemented in the emmeans package.

### 2.7. Ethical Approval

The study protocol was approved by the Commission for the Regulation of Experimental Research (Bioethics Commission) of the Severtsov Institute of Ecology and Evolution, Russian Academy of Sciences (protocol code No. 21, date of approval: 24 April 2018). The procedures performed had no obvious adverse effects on the animals.

## 3. Results

### 3.1. fGCm Concentrations

**Figure 1:**
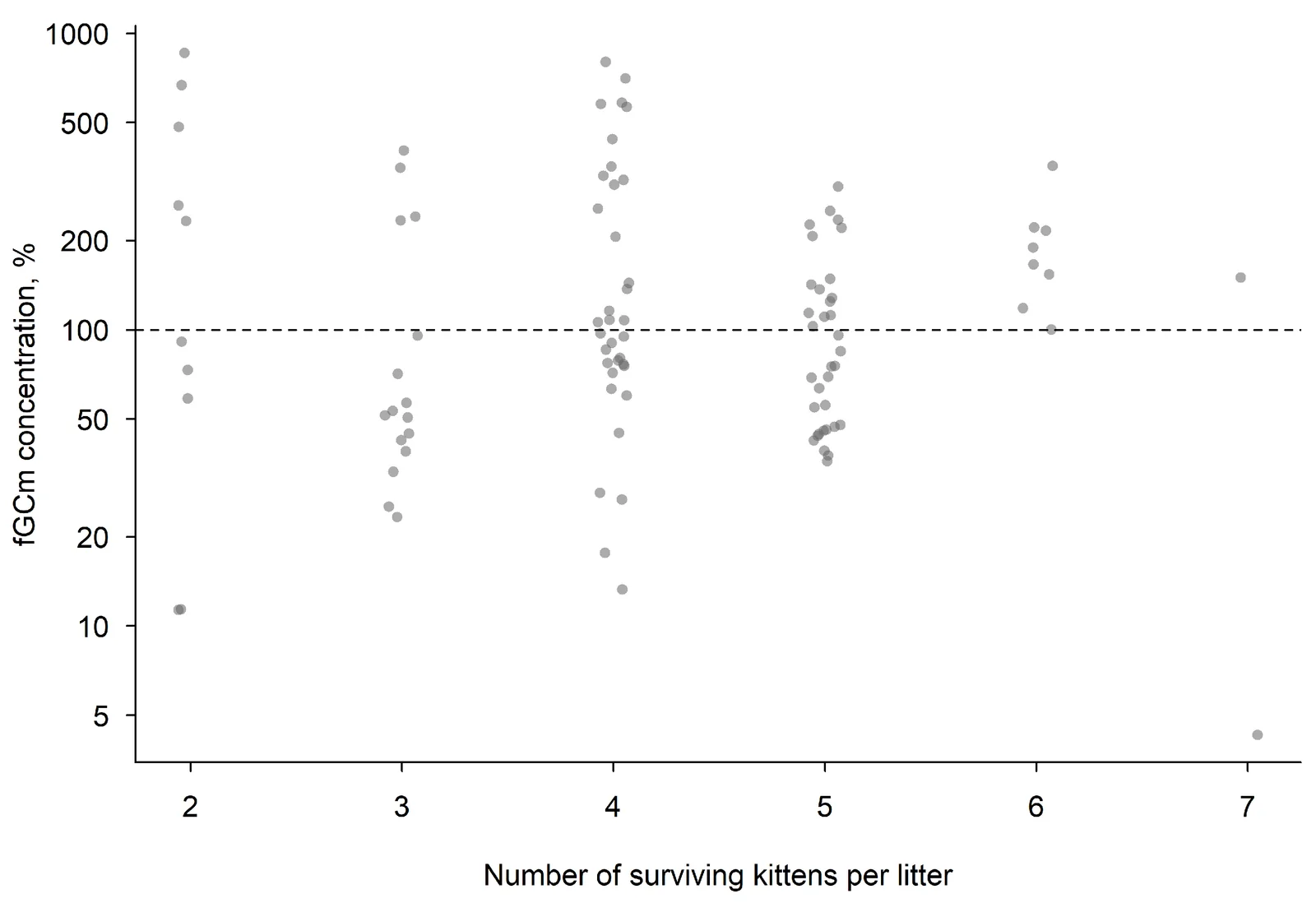
Relationship between relative fGCm concentrations and the number of surviving kittens. Points represent individual faecal samples. The dashed horizontal line indicates the individual baseline level (100%). The y-axis is presented on a logarithmic scale.

fGCm concentrations in domestic cats ranged from 39 to 3725 ng/g of dry faeces (Table S1, Figure S1). Relative fGCm levels during lactation were negatively associated with the number of surviving kittens, with females having fewer surviving kittens exhibiting higher fGCm concentrations (Estimate = −0.382 ± 0.101 SE; *p* < 0.001) (Figure 1, Table S2). Litter losses were not included in any of the models within ΔAICc < 2.

**Figure 2:**
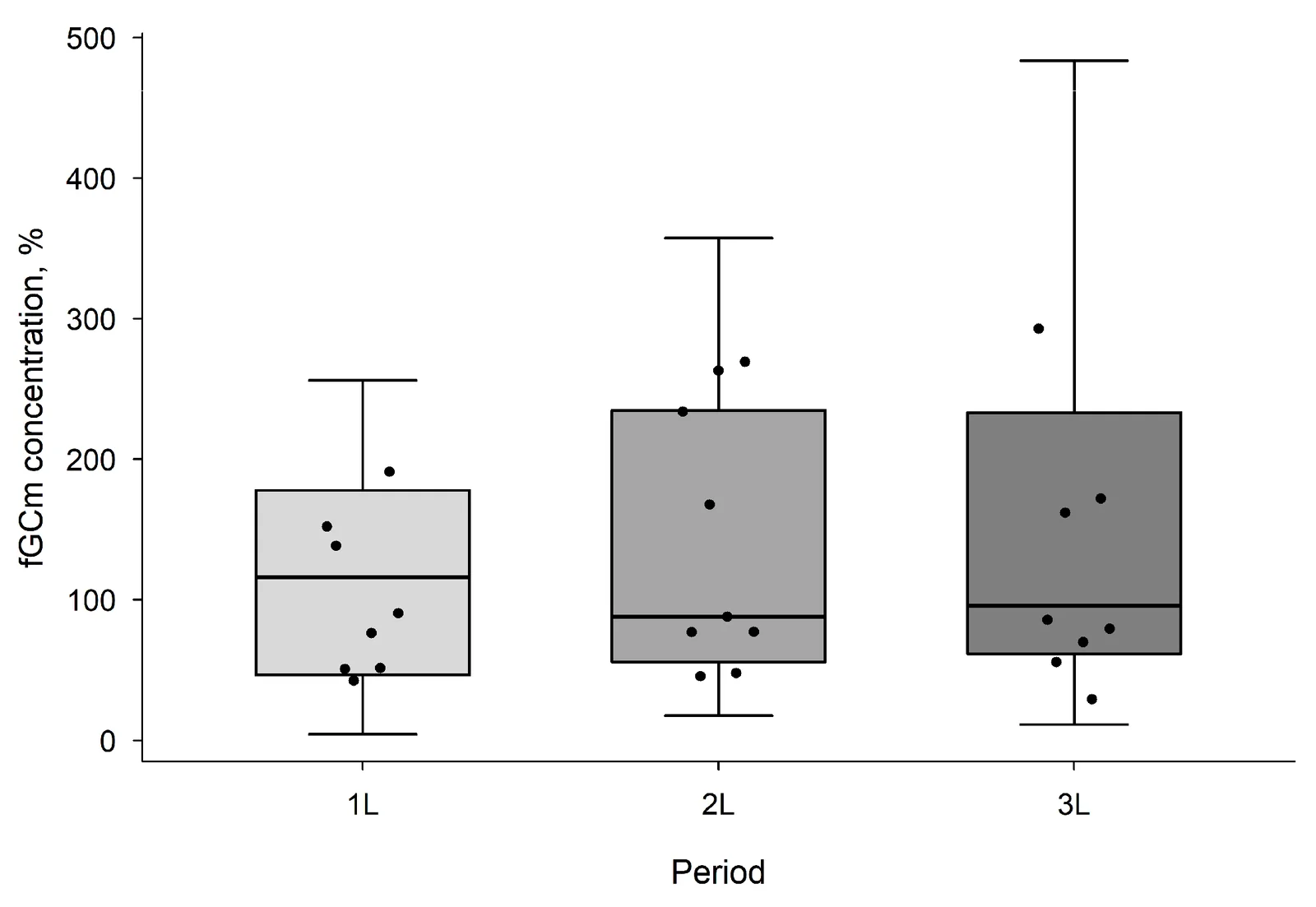
Relative fGCm concentrations in female domestic cats (in %) for each study period. The x-axis represents the periods: first (1 L), second (2 L), and third (3 L) months of lactation. The y-axis represents fGCm concentrations in percentages. The horizontal line within each box represents the median, the lower and upper borders represent the first and third quartiles, and the whiskers extend to the smallest and largest observations within 1.5 times the interquartile range. For each female and study period, the median concentration across all available faecal samples was calculated. Individual female-specific monthly medians are shown as black points (*n* = 9 per period).

Lactation period had no significant effect on relative fGCm concentrations (LMM: F_2_,94 = 0.23; *p* = 0.791) (Figure 2). Consistently, post hoc comparisons revealed no differences between the first, second, and third months of lactation (Tukey-adjusted *p* = 0.916 for first vs. second month; *p* = 0.775 for first vs. third month, and *p* = 0.960 for second vs. third month).

Individual median baseline fGCm concentrations were not associated with reproductive parameters. No significant relationships were found between baseline fGCm levels and kitten survival to the end of the first month of lactation (Binomial Model: Estimate = 0.0015 ± 0.0012 SE; *p* = 0.203) or litter size at birth (Linear Model: Estimate = 0.269 ± 0.934 SE; *p* = 0.782).

### 3.2. fPm Concentrations

fPm concentrations in female domestic cats ranged from 52 to 34,497 ng/g dry faeces during the study period (Table S1, Figure S2). The positive association between relative fPm concentrations and the number of surviving kittens did not reach statistical significance (Estimate = 0.208 ± 0.118 SE; *p* = 0.078). No significant effect of litter losses was found (*p* = 0.447) (Table S2).

**Figure 3:**
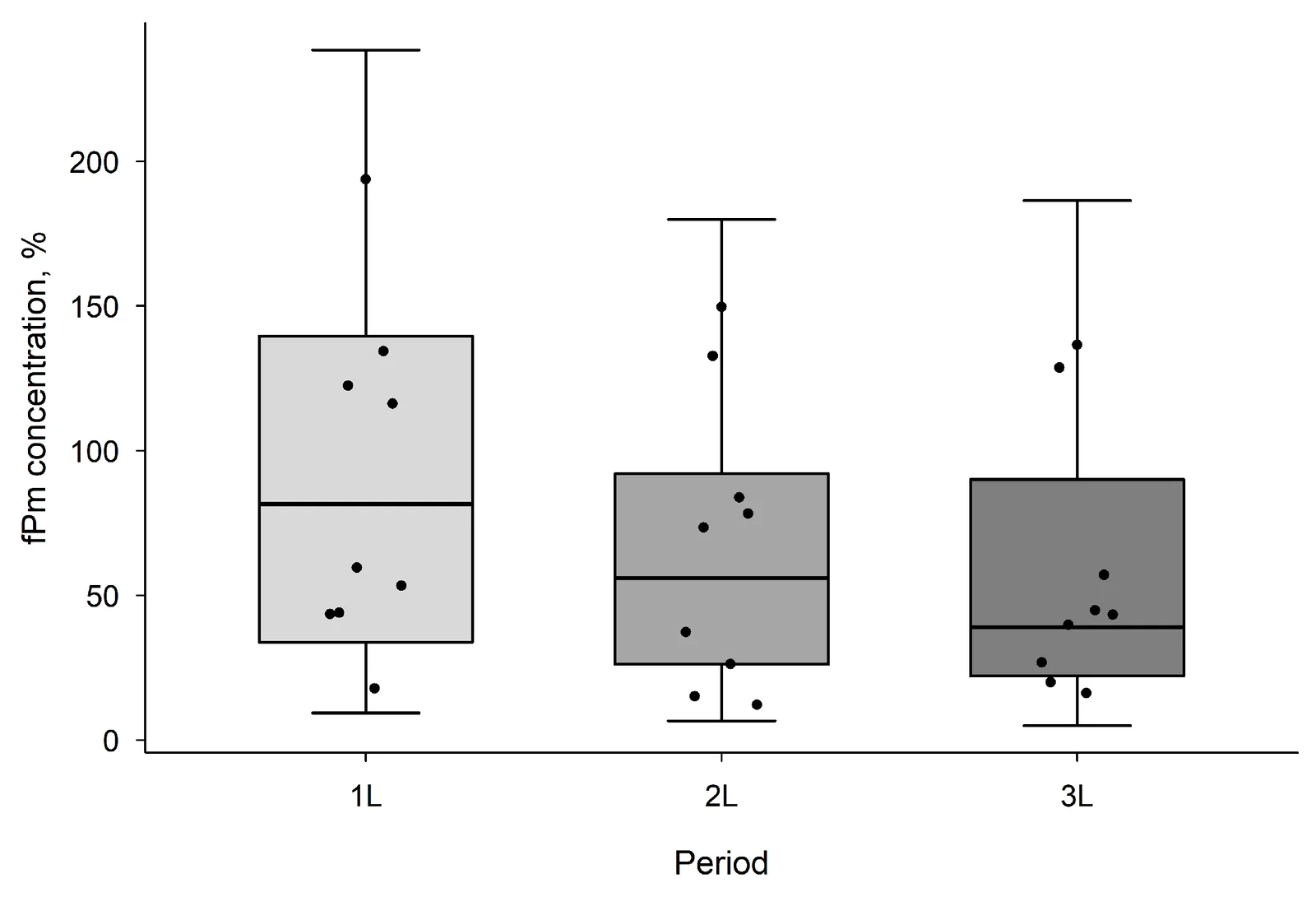
Relative fPm concentrations in female domestic cats (in %) for each study period. The x-axis represents the periods: first (1 L), second (2 L), and third (3 L) months of lactation. The y-axis represents fPm concentrations in percentages. The horizontal line within each box represents the median, the lower and upper borders represent the first and third quartiles, and the whiskers extend to the smallest and largest observations within 1.5 times the interquartile range. For each female and study period, the median concentration across all available faecal samples was calculated. Individual female-specific monthly medians are shown as black points (*n* = 9 per period).

Lactation period had a significant effect on relative fPm concentrations (LMM: F_2_,94 = 4.49; *p* = 0.014) (Figure 3). Post hoc comparisons revealed that fPm levels in the third month of lactation were significantly lower than in the first month (Tukey-adjusted *p* = 0.012), whereas no significant differences were found between the first and second months (*p* = 0.103) or between the second and third months (*p* = 0.698).

Individual median baseline fPm concentrations were not associated with reproductive parameters. No significant relationships were found between baseline fPm levels and kitten survival to the end of the first month of lactation (Binomial Model: Estimate = 0.0008 ± 0.0010 SE; *p* = 0.396) or litter size at birth (*p* = 0.739).

### 3.3. fTm Concentrations

fTm concentrations in female domestic cats ranged from 89 to 6360 ng/g of dry faeces during the study period (Table S1, Figure S3). Relative fTm concentrations were not associated with the number of surviving kittens (Estimate = 3.625 ± 3.123 SE; *p* = 0.246) or litter losses (*p* = 0.603) (Table S2).

**Figure 4:**
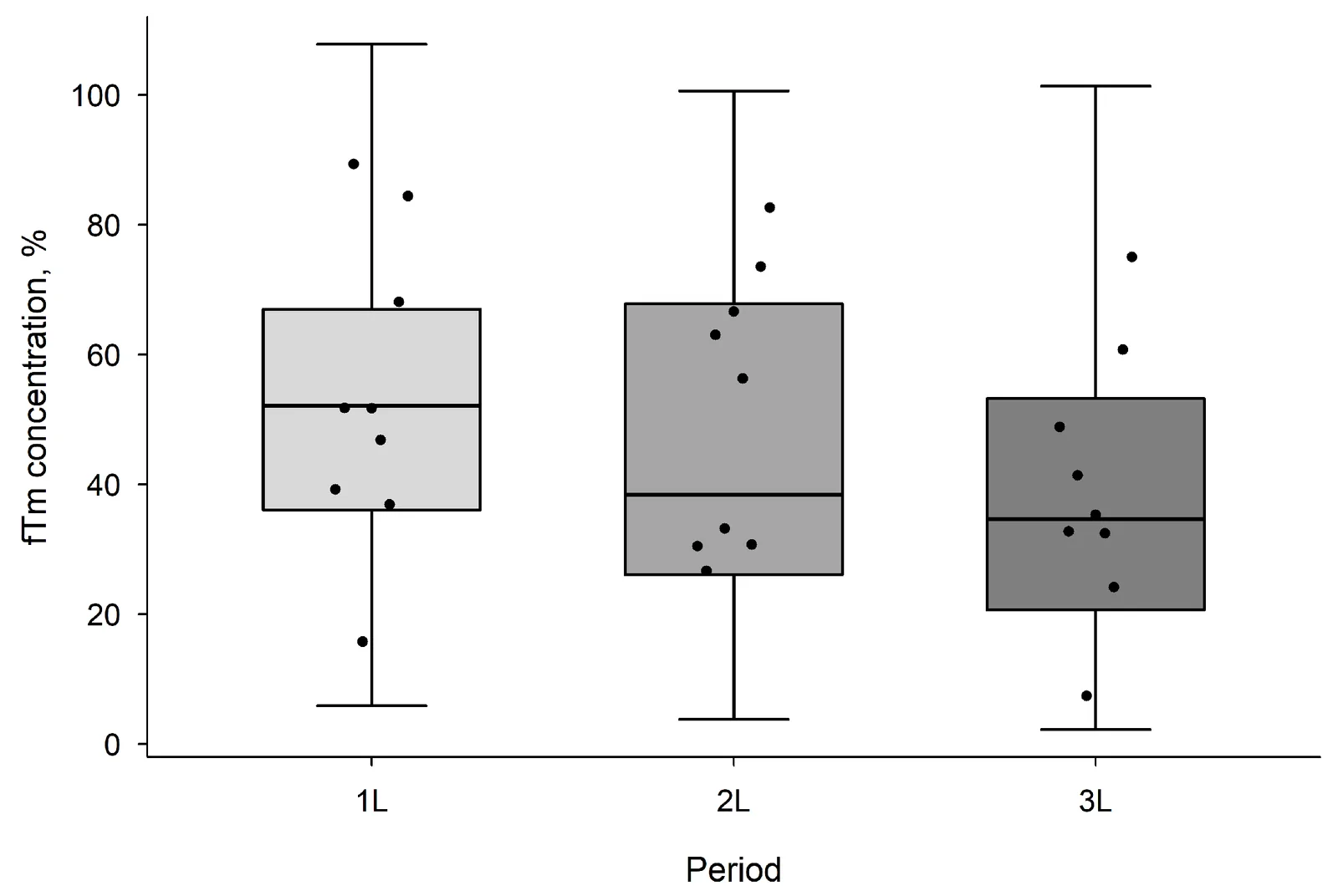
Relative fTm concentrations in female domestic cats (in %) for each study period. The x-axis represents the periods: first (1 L), second (2 L), and third (3 L) months of lactation. The y-axis represents fTm concentrations in percentages. The horizontal line within each box represents the median, the lower and upper borders represent the first and third quartiles, and the whiskers extend to the smallest and largest observations within 1.5 times the interquartile range. For each female and study period, the median concentration across all available faecal samples was calculated. Individual female-specific monthly medians are shown as black points (*n* = 9 per period).

Lactation period showed a marginal but non-significant effect on relative fTm concentrations (LMM: F_2_,94 = 2.90; *p* = 0.060). However, fTm concentrations tended to decrease over the course of lactation and were lower in the third month than in the first month (Estimate = −14.22 ± 5.91 SE; Tukey-adjusted *p* = 0.047) (Figure 4). No significant differences were found between the first and second months or between the second and third months (Tukey-adjusted *p* = 0.496 and *p* = 0.430, respectively).

Individual median baseline fTm concentrations were negatively associated with kitten survival to the end of the first month, although this relationship did not reach statistical significance (Binomial Model: Estimate = −1.831 ± 0.976 SE; *p* = 0.061). Two females with the highest individual median baseline fTm concentrations (Sirena, 4804.5 ng/g and Merida, 6052.8 ng/g dry faeces) had the lowest numbers of kittens surviving to the end of the first month (3 and 2 kittens, respectively). A moderate negative correlation was observed between individual median baseline fTm concentrations and the proportion of kittens surviving to the end of the first month (Pearson’s correlation r = −0.61; *p* = 0.078). No significant relationship was found between baseline fTm concentrations and litter size at birth (*p* = 0.563).

### 3.4. Associations Among Hormone Concentrations

Model-based analyses consistent positive associations among all studied steroid hormone groups during lactation. Relative fGCm concentrations were positively associated with relative fPm (Estimate = 0.277 ± 0.101 SE; *p* = 0.006). The association between relative fGCm and relative fTm concentrations was positive but did not reach statistical significance (Estimate = 5.446 ± 2.911 SE; *p* = 0.061). Relative fPm concentrations were positively associated with fTm concentrations (Estimate = 0.0122 ± 0.003 SE, *p* < 0.001).

## 4. Discussion

During lactation, a female’s body undergoes substantial physiological changes, including alterations in steroid hormone concentrations [34]. The dynamics of these hormones vary considerably among species [35,36] and remain poorly studied in members of the Felidae family. Lactation is associated with high energetic demands on the maternal organism [37]; therefore, we expected fGCm levels to be elevated during lactation and to vary across lactation stages. However, relative fGCm concentrations did not differ among the first, second, and third months of lactation. In domestic cats, kittens begin consuming solid food towards the end of the fourth week of life, and maternal energetic costs associated with milk production are expected to peak during this period [1]. In line with this, fGCm levels during the first month of lactation were slightly elevated, reaching approximately 116% of baseline values. This may reflect maximal mobilisation of maternal resources for milk production when kittens are still fully dependent on maternal nutrition [38]. It is worth noting that differences between our study and that of Alekseeva et al. (2020) [1] may arise from sampling design: while their work focused on the end of the first month, when energetic demands peak, our data cover the entire first month, including early stages when maternal investment is still relatively lower.

From the second month of lactation, kittens progressively switch to solid food [39], which reduces the physiological load on the female. Accordingly, we expected fGCm concentrations to decrease after the first month. However, this pattern was not observed: relative fGCm concentrations did not decrease significantly across lactation stages. This lack of decline may be explained by the role of glucocorticoids during lactation [40]. Transient increases in GC levels during nursing have been reported in some mammalian species [41,42,43] and are thought to be associated with energy mobilisation supporting milk production. Glucocorticoids are involved in the regulation of mammary gland function, including gene expression in mammary epithelial cells and maintenance of lactation [44]. Their levels may remain relatively stable throughout the lactation period as part of normal physiological regulation [45] rather than reflecting stress alone. The absence of a decline may also reflect behavioural factors, such as increased activity of growing kittens and their frequent interactions with mothers in confined conditions.

Suckling stimulation contributes to the neuroendocrine regulation of the maternal HPA axis during lactation. More frequent stimulation of the nipples promotes the release of oxytocin and prolactin, which can attenuate HPA axis responsiveness and suppress glucocorticoid secretion to acute stress [44,46,47]. Variation in suckling intensity associated with litter size may influence maternal oxytocin release and contribute to HPA axis hyporesponsiveness, potentially resulting in greater attenuation of cortisol secretion in females rearing larger litters. Although this hypothesis remains to be confirmed in domestic cats, it provides a biologically plausible framework for interpreting the patterns identified here. Litter losses were not associated with relative fGCm concentrations; therefore, the observed relationship does not indicate that increased glucocorticoid secretion caused kitten mortality or reduced litter size during lactation.

Although progesterone is primarily associated with the maintenance of pregnancy, luteal progesterone secretion in felids may continue after parturition. In domestic cats, the corpora lutea persist until the fourth week of lactation, after which they begin to regress [48]. The postpartum activity of the corpus luteum is likely regulated by prolactin, whose luteotropic function has been described in several carnivore species [49,50,51], including the domestic cat [52]. In the present study, relative fPm concentrations were lower during the third than during the first month of lactation, whereas no significant differences were found between the first and second or the second and third months. Because the first samples were collected 1–6 days after parturition, some of the earliest measurements may have partly reflected the endocrine state around parturition and contributed to the higher values observed in the first month. However, the analysis included all samples collected weekly throughout each month, so the higher fPm concentrations during the first month cannot be attributed solely to the timing of the earliest postpartum samples. This pattern may be consistent with a gradual decline in luteal activity as lactation progresses rather than an abrupt reduction immediately after the first month. Prolonged persistence of luteal tissue has also been described in other felids. For example, in the Eurasian lynx (*Lynx lynx*), the life cycle of the corpus luteum includes the persistence of steroidogenic luteal cells throughout the year, with both fully functional and luteolytic histological features observed simultaneously in the ovaries [48]. With respect to reproductive outcomes, relative fPm concentrations were not significantly associated with either the number of surviving kittens or litter losses, suggesting that their postpartum decline primarily reflected changes in luteal activity rather than a direct relationship with reproductive success in this study.

Glucocorticoids, progesterone and androgens are derived from a common steroidogenic pathway beginning with cholesterol, with pregnenolone acting as a key intermediate [53,54]. In this context, the positive associations of relative fPm concentrations with relative fGCm and fTm concentrations may reflect shared steroidogenic pathways and coordinated endocrine regulation. The positive relationship between relative fGCm and fTm concentrations, although not statistically significant, may also be consistent with shared adrenal regulation. In female mammals, ACTH stimulates adrenal steroidogenesis, with DHEA and androstenedione representing the major adrenal androgens and serving as precursors for peripheral testosterone synthesis [4]. This ACTH-dependent pathway may therefore contribute to the relationship between relative fGCm and fTm concentrations observed in our study.

Parental behaviour in animals largely depends on the influence of hormones [55], which can either hinder or, conversely, enhance parental care. In males, elevated androgen levels are generally associated with increased investment in mating behaviour and reduced paternal care [56], whereas lower testosterone concentrations are often linked to increased paternal behaviour [57]. The role of androgens in the maternal physiology of female carnivores remains poorly understood. In domestic cats, studies of testosterone dynamics have focused mainly on males and prepubertal individuals [58,59,60], whereas the influence of androgens on maternal physiology in females of this species remains limited [15]. In the present study, median baseline fTm concentrations showed a negative relationship with kitten survival to the end of the first month, although this relationship did not reach statistical significance in either the binomial model or the correlation analysis. This pattern was also evident in the individual data: the two females with the highest median baseline fTm concentrations had the lowest numbers of kittens surviving to the end of the first month. Together, these results suggest a possible relationship between the baseline androgen status of females and early offspring survival, although the underlying mechanisms require further investigation. The effects of maternal testosterone on offspring growth and survival have been investigated mainly in the context of hormonal changes during pregnancy [61,62,63], although endocrine conditions established before mating may also have long-term consequences for offspring viability.

The lower relative fTm concentrations observed during the third compared with the first month of lactation may have implications for oestrogen synthesis. Oestrogens, especially 17β-oestradiol, the most biologically active form of oestrogen synthesized from testosterone, contribute to the regulation of maternal behaviour [6]. Therefore, changes in androgen concentrations during lactation could potentially influence maternal physiology not only directly, but also indirectly through altered oestrogen production. A parallel pattern of testosterone and oestrogen dynamics during lactation has previously been described in New Zealand white rabbits [64]. Although oestrogen metabolites were not assessed in the present study, such endocrine changes may contribute to gradual shifts in maternal responsiveness over the course of lactation. Future studies combining measurements of androgen and oestrogen metabolites with assessments of maternal behaviour would provide a more complete understanding of endocrine changes during lactation in domestic cats.

We expected females with higher glucocorticoid concentrations to have larger litters because of the high energetic demands associated with rearing offspring. The results only partially supported this expectation: relative fGCm concentrations were associated with the number of surviving kittens, but the relationship was negative rather than positive, and no association with litter losses was found. Baseline fGCm concentrations were unrelated to reproductive outcomes. This inverse relationship may reflect stronger suckling-induced attenuation of HPA axis activity in females rearing more kittens. For progesterone, only the temporal component of our prediction was supported. Relative fPm concentrations decreased from the first to the third month of lactation, consistent with the gradual regression of the corpora lutea, but were not associated with the number of surviving kittens or litter losses. Finally, we expected higher testosterone concentrations to be associated with lower offspring survival and increased litter losses. This relationship was not statistically confirmed, as neither relative fTm concentrations during lactation nor baseline fTm concentrations were significantly associated with kitten survival or litter losses. However, the relationship between baseline fTm concentrations and kitten survival was negative, and the two females with the highest median baseline fTm concentrations had the lowest numbers of surviving kittens.

## 5. Conclusions

The three steroid metabolite groups showed distinct relationships with reproductive parameters in female domestic cats. Relative fGCm concentrations during lactation were negatively associated with the number of surviving kittens, although they were not associated with litter losses. This pattern may reflect variation in suckling stimulation and energetic demands rather than a direct effect of glucocorticoids on kitten mortality. Relative fPm concentrations declined during lactation but were not associated with either the number of surviving kittens or litter losses, suggesting that their dynamics primarily reflected postpartum changes in luteal activity. Relative fTm concentrations were lower during the third than during the first month of lactation but were not associated with either the number of surviving kittens or litter losses. Median baseline fTm concentrations showed a negative relationship with kitten survival, although this relationship did not reach statistical significance. Further longitudinal studies using larger datasets and incorporating repeated endocrine measurements, assessments of maternal behaviour, and indicators of kitten condition are needed to clarify the broader role of maternal endocrine mechanisms in offspring development and survival.

## Figures and Tables

**Table 1:** Females used in the study with total number of kittens born (including stillborn kittens); number of kittens surviving by the end of the first, second, and third months of lactation; and mean kitten body mass in the litter in the first days of life and by the end of the first, second, and third months of lactation.

Female ID	Number of Kittens per Litter at Different Lactation Stages (in Months)				Mean Body Mass of a Kitten at Different Lactation Stages (in Months), kg			
	0	1	2	3	0	1	2	3
Astrid	7	6	3	3	0.101	0.171	0.4	0.567
Cleo	5	5	5	5	0.11	0.285	0.617	0.841
Fishka	5	5	5	5	0.105	0.249	0.527	0.769
Merida	5	2	2	2	0.103	0.375	0.878	1.165
Milka	4	4	4	4	0.134	0.358	0.516	0.865
Riska	4	4	4	4	0.118	0.336	0.631	0.904
Sirena	4	3	3	3	0.125	0.32	0.737	1.048
Torvi	8	6	5	5	0.086	0.227	0.607	0.828
Yolka	5	4	4	4	0.109	0.305	0.683	0.919

## Data Availability

The data presented in this study are available from the corresponding author upon reasonable request.

## Supplementary Materials

The following supporting information can be downloaded at https://www.mdpi.com/article/10.3390/ani16162523/s1. Table S1: Individual absolute concentrations of faecal steroid metabolites and reproductive parameters in female domestic cats (n = 9) during lactation (first (1 L), second (2 L), and third (3 L) month). Table S2: Individual relative concentrations of faecal steroid metabolites and reproductive parameters in female domestic cats (n = 9) during lactation (first (1L), second (2 L), and third (3 L) month). Figure S1: Absolute fGCm concentrations (ng/g) in female domestic cats during each study period. The x-axis represents the first (1 L), second (2 L), and third (3 L) months of lactation. The y-axis represents absolute fGCm concentrations. The horizontal line within each box represents the median, the lower and upper borders represent the first and third quartiles, and the whiskers extend to the smallest and largest observations within 1.5 times the interquartile range. For each female and study period, the median concentration across all available samples was calculated. Female-specific monthly medians are shown as black points (n = 9 per period). Figure S2: Absolute fPm concentrations (ng/g) in female domestic cats during each study period. The x-axis represents the first (1 L), second (2 L), and third (3 L) months of lactation. The y-axis represents absolute fPm concentrations. The horizontal line within each box represents the median, the lower and upper borders represent the first and third quartiles, and the whiskers extend to the smallest and largest observations within 1.5 times the interquartile range. For each female and study period, the median concentration across all available samples was calculated. Female-specific monthly medians are shown as black points (n = 9 per period). Figure S3: Absolute fTm concentrations (ng/g) in female domestic cats during each study period. The x-axis represents the first (1 L), second (2 L), and third (3 L) months of lactation. The y-axis represents absolute fTm concentrations. The horizontal line within each box represents the median, the lower and upper borders represent the first and third quartiles, and the whiskers extend to the smallest and largest observations within 1.5 times the interquartile range. For each female and study period, the median concentration across all available samples was calculated. Female-specific monthly medians are shown as black points (n = 9 per period).
