# Supplementary material for: Faecal Steroid Metabolite Profiles and Reproductive Outcomes in Domestic Cat (Felis catus)
Source: Animals (Basel). 2026 Aug 13;16(16):2523. doi: 10.3390/ani16162523 (PMC13508889; doi:10.3390/ani16162523)
Supplement: Supplementary file 1 [file animals-16-02523-s001.zip › animals-4419308-supplementary.pdf]

Supplementary Materials.

Table S1. Individual absolute concentrations of faecal steroid metabolites and reproductive parameters in female domestic cats (n = 9) during lactation (first (1 L), second (2 L) and third (3 L) month).

ID	Cortisol, ng/g	Progesterone, ng/g	Testosterone, ng/g	Period	Number of surviving kittens	Litter losses	Week of lactation
Astrid	1391	585	1245	1L	7	0.1	0
Astrid	40	711	349	1L	7	0.1	0
Astrid	1755	1135	1833	1L	6	0.2	1
Astrid	1425	207	584	1L	6	0.2	0
Astrid	928	449	608	2L	6	0.2	0
Astrid	2099	662	813	2L	5	0.2	1
Astrid	2862	99	89	2L	4	0.3	1
Astrid	2232	850	1329	2L	3	0.3	1
Astrid	3725	931	1453	3L	3	0.4	0
Astrid	2165	393	922	3L	3	0.5	0
Astrid	886	405	1137	3L	3	0.5	0
Astrid	3258	124	1140	3L	3	0.6	0
Yolka	1168	1160	1890	1L	5	0.2	0
Yolka	376	259	342	1L	5	0.2	0
Yolka	720	1355	1599	1L	5	0.2	0
Yolka	1434	393	904	1L	4	0.3	1
Yolka	184	279	118	2L	4	0.4	0
Yolka	1108	178	1226	2L	4	0.5	0
Yolka	805	163	1479	2L	4	0.7	0
Yolka	1503	405	1273	3L	4	0.7	0
Yolka	627	858	2095	3L	4	0.7	0
Yolka	839	259	952	3L	4	0.8	0
Yolka	822	1008	318	3L	4	0.9	0
Cleo	2026	816	1617	1L	5	0.2	0
Cleo	1363	1348	1874	1L	5	0.2	0
Cleo	1175	640	1402	1L	5	0.3	0
Cleo	422	211	718	1L	5	0.3	0
Cleo	636	265	1392	2L	5	0.3	0
Cleo	402	146	279	2L	5	0.4	0
Cleo	941	676	672	2L	5	0.4	0
Cleo	775	235	884	2L	5	0.6	0
Cleo	879	1541	1523	3L	5	0.6	0
Cleo	689	466	1038	3L	5	0.7	0
Cleo	692	191	360	3L	5	0.8	0
Cleo	1044	192	872	3L	5	0.8	0
Merida	248	8534	3001	1L	4	0.2	1
Merida	46	347	356	1L	4	0.2	0
Merida	39	113	593	1L	2	0.3	2
Merida	2977	1087	1313	1L	2	0.4	0
Merida	2317	3314	2525	2L	2	0.5	0

Merida	909	1541	1881	2L	2	0.5	0
Merida	253	1652	2139	2L	2	0.7	0
Merida	203	898	1736	2L	2	0.8	0
Merida	805	400	704	3L	2	0.9	0
Merida	1674	1448	1813	3L	2	0.9	0
Merida	39	543	195	3L	2	1.1	0
Merida	316	414	183	3L	2	1.2	0
Milka	1281	897	922	1L	4	0.2	0
Milka	2338	1188	1565	1L	4	0.3	0
Milka	2055	799	1640	1L	4	0.3	0
Milka	746	1033	1201	1L	4	0.4	0
Milka	1035	1788	2692	2L	4	0.4	0
Milka	933	896	1751	2L	4	0.4	0
Milka	283	371	408	2L	4	0.4	0
Milka	600	595	1815	2L	4	0.5	0
Milka	964	287	807	3L	4	0.5	0
Milka	1683	1815	900	3L	4	0.7	0
Milka	1699	929	1277	3L	4	0.8	0
Milka	1646	432	990	3L	4	0.9	0
Riska	964	7637	1129	1L	4	0.2	0
Riska	373	303	836	1L	4	0.3	0
Riska	234	413	1428	1L	4	0.3	0
Riska	898	278	900	1L	4	0.3	0
Riska	713	527	1601	2L	4	0.4	0
Riska	628	454	579	2L	4	0.5	0
Riska	750	737	841	2L	4	0.5	0
Riska	788	524	1741	2L	4	0.6	0
Riska	895	418	951	3L	4	0.6	0
Riska	635	335	456	3L	4	0.7	0
Riska	524	317	1176	3L	4	0.8	0
Riska	222	99	214	3L	4	0.9	0
Sirena	763	6751	1694	1L	3	0.2	1
Sirena	601	770	2280	1L	3	0.3	0
Sirena	692	1347	1772	1L	3	0.3	0
Sirena	570	923	1822	2L	3	0.4	0
Sirena	717	758	1108	2L	3	0.5	0
Sirena	957	1004	1202	2L	3	0.6	0
Sirena	524	551	1750	2L	3	0.7	0
Sirena	314	1228	995	3L	3	0.7	0
Sirena	341	223	1324	3L	3	0.8	0
Sirena	447	519	1533	3L	3	0.9	0
Sirena	681	380	106	3L	3	1.0	0
Torvi	1289	922	1601	1L	6	0.1	2
Torvi	1673	1139	1654	1L	6	0.2	0
Torvi	917	948	3069	1L	6	0.2	0
Torvi	1716	391	1622	1L	6	0.2	0
Torvi	2770	1377	1946	2L	6	0.3	0
Torvi	2354	543	1111	2L	5	0.3	1

Torvi	1819	716	1940	2L	5	0.4	0
Torvi	328	384	1599	2L	5	0.5	0
Torvi	1605	733	1969	3L	5	0.6	0
Torvi	965	203	350	3L	5	0.7	0
Torvi	1060	715	2438	3L	5	0.7	0
Torvi	1951	176	953	3L	5	0.8	0
Fishka	674	1843	969	1L	5	0.1	0
Fishka	830	1438	2427	1L	5	0.2	0
Fishka	713	5705	2049	1L	5	0.2	0
Fishka	2157	1346	2489	1L	5	0.2	0
Fishka	692	1108	2203	2L	5	0.3	0
Fishka	845	985	1579	2L	5	0.3	0
Fishka	571	482	629	2L	5	0.4	0
Fishka	963	2176	1558	3L	5	0.5	0
Fishka	1679	3095	2020	3L	5	0.5	0
Fishka	725	535	514	3L	5	0.7	0
Fishka	593	1273	307	3L	5	0.8	0

Table S2. Individual relative concentrations of faecal steroid metabolites and reproductive parameters in female domestic cats (n = 9) during lactation (first (1 L), second (2 L) and third (3 L) month).

ID	Cortisol, %	Progesterone, %	Testosterone, %	Period	Number of surviving kittens	Litter losses	Week of lactation
Astrid	150	39	53	1L	7	0.1	0
Astrid	4.3	48	15	1L	7	0.1	0
Astrid	190	76	79	1L	6	0.2	1
Astrid	154	14	25	1L	6	0.2	0
Astrid	100	30	26	2L	6	0.2	0
Astrid	227	45	35	2L	5	0.2	1
Astrid	309	6.7	3.8	2L	4	0.3	1
Astrid	241	57	57	2L	3	0.3	1
Astrid	402	63	62	3L	3	0.4	0
Astrid	234	26	40	3L	3	0.5	0
Astrid	96	27	49	3L	3	0.5	0
Astrid	352	8.3	49	3L	3	0.6	0
Yolka	112	80	127	1L	5	0.2	0
Yolka	36	18	23	1L	5	0.2	0
Yolka	69	93	108	1L	5	0.2	0
Yolka	137	27	61	1L	4	0.3	1
Yolka	18	19	7.9	2L	4	0.4	0
Yolka	106	12	83	2L	4	0.5	0
Yolka	77	11	100	2L	4	0.7	0
Yolka	144	28	86	3L	4	0.7	0
Yolka	60	59	141	3L	4	0.7	0

Yolka	80	18	64	3L	4	0.8	0
Yolka	79	69	21	3L	4	0.9	0
Cleo	221	49	55	1L	5	0.2	0
Cleo	149	82	64	1L	5	0.2	0
Cleo	128	39	48	1L	5	0.3	0
Cleo	46	13	25	1L	5	0.3	0
Cleo	69	16	48	2L	5	0.3	0
Cleo	44	8.8	10	2L	5	0.4	0
Cleo	103	41	23	2L	5	0.4	0
Cleo	85	14	30	2L	5	0.6	0
Cleo	96	93	52	3L	5	0.6	0
Cleo	75	28	36	3L	5	0.7	0
Cleo	75	12	12	3L	5	0.8	0
Cleo	114	12	30	3L	5	0.8	0
Merida	72	710	50	1L	4	0.2	1
Merida	13	29	5.9	1L	4	0.2	0
Merida	11	9.4	10	1L	2	0.3	2
Merida	860	90	22	1L	2	0.4	0
Merida	669	276	42	2L	2	0.5	0
Merida	263	128	31	2L	2	0.5	0
Merida	73	137	35	2L	2	0.7	0
Merida	59	75	29	2L	2	0.8	0
Merida	233	33	12	3L	2	0.9	0
Merida	484	120	30	3L	2	0.9	0
Merida	11	45	3.2	3L	2	1.1	0
Merida	91	34	3.0	3L	2	1.2	0
Milka	440	180	34	1L	4	0.2	0
Milka	802	239	59	1L	4	0.3	0
Milka	705	160	61	1L	4	0.3	0
Milka	256	208	45	1L	4	0.4	0
Milka	355	359	101	2L	4	0.4	0
Milka	320	180	65	2L	4	0.4	0
Milka	97	74	15	2L	4	0.4	0
Milka	206	119	68	2L	4	0.5	0
Milka	331	58	30	3L	4	0.5	0
Milka	578	365	34	3L	4	0.7	0
Milka	583	187	48	3L	4	0.8	0
Milka	565	87	37	3L	4	0.9	0
Riska	116	381	52	1L	4	0.2	0
Riska	45	15	39	1L	4	0.3	0
Riska	28	21	66	1L	4	0.3	0
Riska	108	14	42	1L	4	0.3	0
Riska	86	26	74	2L	4	0.4	0
Riska	76	23	27	2L	4	0.5	0
Riska	90	37	39	2L	4	0.5	0
Riska	95	26	80	2L	4	0.6	0
Riska	108	21	44	3L	4	0.6	0
Riska	76	17	21	3L	4	0.7	0

Riska	63	16	54	3L	4	0.8	0
Riska	27	4.9	10	3L	4	0.9	0
Sirena	57	674	35	1L	3	0.2	1
Sirena	45	77	47	1L	3	0.3	0
Sirena	51	134	37	1L	3	0.3	0
Sirena	42	92	38	2L	3	0.4	0
Sirena	53	76	23	2L	3	0.5	0
Sirena	71	100	25	2L	3	0.6	0
Sirena	39	55	36	2L	3	0.7	0
Sirena	23	123	21	3L	3	0.7	0
Sirena	25	22	28	3L	3	0.8	0
Sirena	33	52	32	3L	3	0.9	0
Sirena	51	38	2.2	3L	3	1.0	0
Torvi	166	115	67	1L	6	0.1	2
Torvi	216	142	69	1L	6	0.2	0
Torvi	118	118	128	1L	6	0.2	0
Torvi	221	49	67	1L	6	0.2	0
Torvi	357	171	81	2L	6	0.3	0
Torvi	304	68	46	2L	5	0.3	1
Torvi	235	89	81	2L	5	0.4	0
Torvi	42	48	66	2L	5	0.5	0
Torvi	207	91	82	3L	5	0.6	0
Torvi	124	25	15	3L	5	0.7	0
Torvi	137	89	101	3L	5	0.7	0
Torvi	252	22	40	3L	5	0.8	0
Fishka	44	138	39	1L	5	0.1	0
Fishka	55	107	97	1L	5	0.2	0
Fishka	47	426	82	1L	5	0.2	0
Fishka	142	100	99	1L	5	0.2	0
Fishka	46	83	88	2L	5	0.3	0
Fishka	56	74	63	2L	5	0.3	0
Fishka	38	36	25	2L	5	0.4	0
Fishka	63	162	62	3L	5	0.5	0
Fishka	111	231	81	3L	5	0.5	0
Fishka	48	40	21	3L	5	0.7	0
Fishka	39	95	12	3L	5	0.8	0

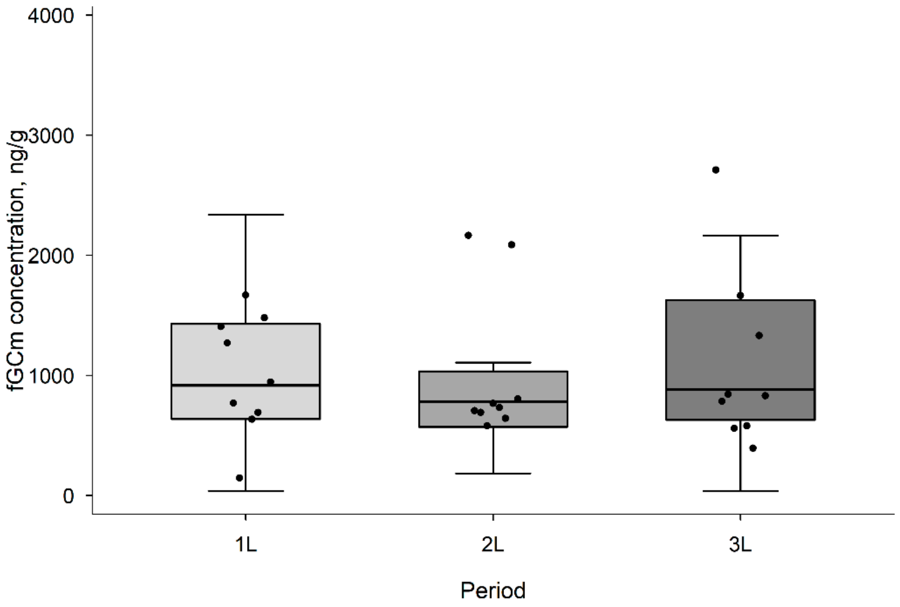

Figure S1. Absolute fGCm concentrations (ng/g) in female domestic cats during each study period. The x-axis represents the first (1 L), second (2 L), and third (3 L) months of lactation. The y-axis represents absolute fGCm concentrations. The horizontal line within each box represents the median, the lower and upper borders represent the first and third quartiles, and the whiskers extend to the smallest and largest observations within 1.5 times the interquartile range. For each female and study period, the median concentration across all available samples was calculated. Female-specific monthly medians are shown as black points (n = 9 per period).

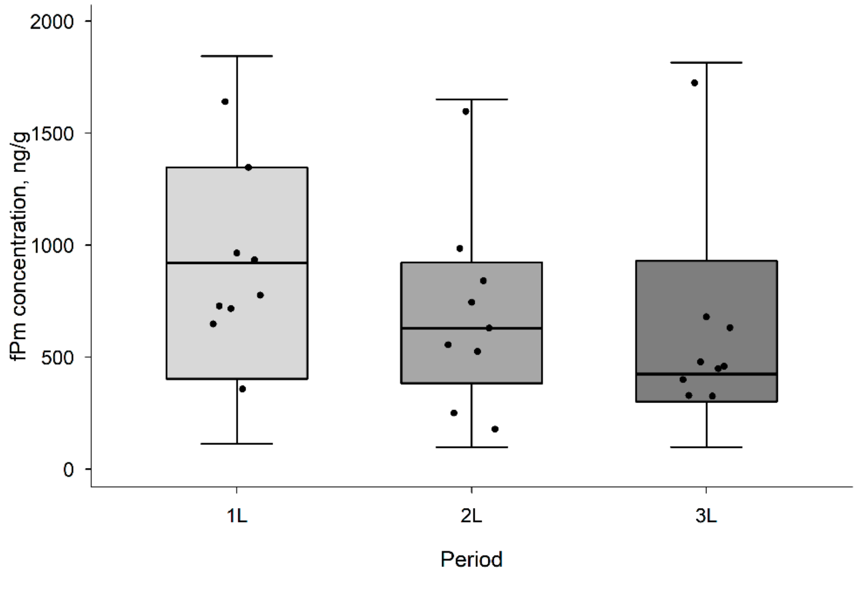

Figure S2. Absolute fPm concentrations (ng/g) in female domestic cats during each study period. The x-axis represents the first (1 L), second (2 L), and third (3 L) months of lactation. The y-axis represents absolute fPm concentrations. The horizontal line within each box represents the median, the lower and upper borders represent the first and third quartiles, and the whiskers extend to the smallest and largest observations within 1.5 times the interquartile range. For each female and study period, the median concentration across all available samples was calculated. Female-specific monthly medians are shown as black points (n = 9 per period).

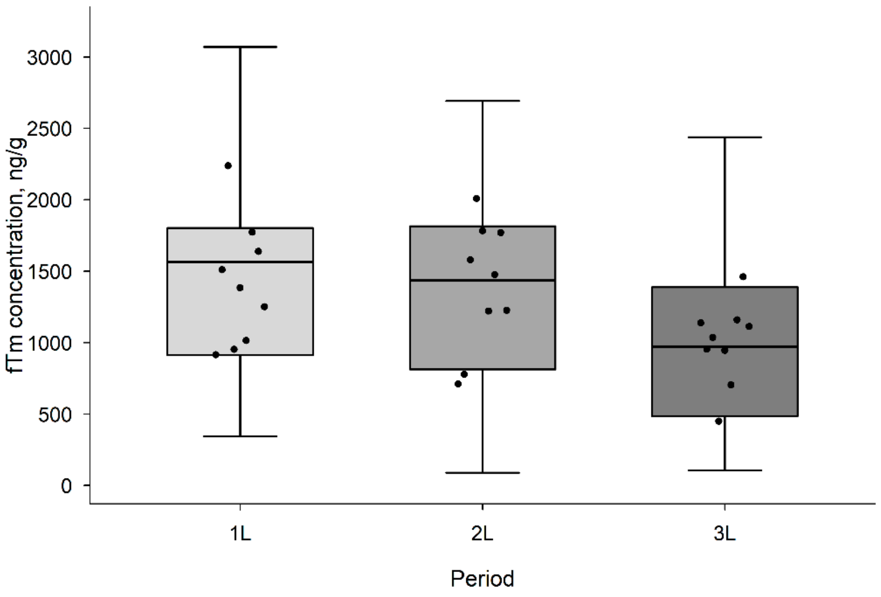

Figure S3. Absolute fTm concentrations (ng/g) in female domestic cats during each study period. The x-axis represents the first (1 L), second (2 L), and third (3 L) months of lactation. The y-axis represents absolute fTm concentrations. The horizontal line within each box represents the median, the lower and upper borders represent the first and third quartiles, and the whiskers extend to the smallest and largest observations within 1.5 times the interquartile range. For each female and study period, the median concentration across all available samples was calculated. Female-specific monthly medians are shown as black points (n = 9 per period).
